# Modification of both functional neurological symptoms and neuroimaging patterns with a good anatomoclinical concordance: a case report

**DOI:** 10.1186/s12883-019-1475-3

**Published:** 2019-11-04

**Authors:** Silvio Galli, Selma Aybek, Sylvie Chokron, Thierry Moulin, Eloi Magnin

**Affiliations:** 1 Department of Neurology, University Hospital of Besançon, 3 bd alexandre fleming, 25030 Besançon, France; 2 Neurology University Clinic, Inselspital, 3010 Bern, Switzerland; 3 Laboratory of Psychology of Perception, UMR 8242, CNRS and Paris Descartes University and Vision and Cognition Unity, Fondation Ophtalmologique Rothschild, Paris, France

**Keywords:** Functional neurological disorders, Conversion disorders, Dissociative disorders, SPECT

## Abstract

**Background:**

In the nineteenth century, Jean Martin Charcot explained functional neurological disorder (formerly called conversion disorder) as a “psychodynamic” lesion. Numerous advances in neuroimaging have permitted identification of the neural underpinnings of this disorder.

**Case presentation:**

Herein we describe a case of functional neurological disorder (FND) with initial left sensorimotor deficit, in-coordinated limb movements, neglect, clouded consciousness, slurred speech and a semiology of visual impairment. A single photon emission computed tomography (SPECT) showed a right thalamic hypoperfusion, which is rather concordant with the initial semiology. Later, the semiology changed, presenting with a predominantly neurovisual complex presentation. The second SPECT showed no more thalamic abnormalities but an hypoperfusion in the right temporo-occipital junction, right inferior parietal lobe and left superior frontal lobe, which is also rather concordant with the changing semiology.

**Conclusions:**

This case illustrates the evolving neuroimaging patterns of FND but also the concordance between semiology and neuroimaging findings in FND supporting Charcot's theory of “dynamic lesion”.

## Background

Functional neurological disorder (FND) is a medical condition in which patients present neurological symptoms not attributed to a known neurological disease. This pathology is a complex entity, as evidenced by the multiple terminologies used (conversion, somatoform, dissociative or somatization disorder) and the various proposed classifications. According to the Diagnostic and statistical manual of mental disorders 5^ed^ (DSM-V), FND is classified among somatic symptoms (formerly called somatoform disorders), whereas in the International classification of diseases 10^ed^ (ICD-10) it is classified among dissociative disorders. These classifications are based on different theoretical models.

Diagnosing this condition in clinical practice is difficult because of a large range of symptom presentation and their fluctuating nature such as sensorimotor deficits, movement disorder, sensorial symptom or psychogenic non-epileptic seizure (PNES). Furthermore, patients can present several symptoms simultaneously with a “chameleon” presentation, as it was already described in the seventeenth century by Sydenham in a patient with a complex semiology [1].

Neuroimaging has allowed a better understanding of the pathophysiological mechanisms of FND, demonstrating potential neural networks associated with clinical signs. Different cortical areas like primary motor cortex in functional motor deficit or visual cortex in functional blindness may show an altered activation or metabolism [2–4]. In addition, other cortical or subcortical structures may simultaneously exhibit abnormal pattern of activation like frontal areas, basal ganglia or hippocampus, in relation to altered emotional and limbic functioning, which in turn may interfer with primary motor or sensory functions (hence the symptom's production).

Here, we present a case of FND with variable semiology, including sensorimotor deficit, unilateral spatial neglect, slurred speech and cortical visual impairments that evolved during the follow-up. Neuroimaging and clinical follow-up reveals a concordance between clinical symptoms and anatomical correlates at the initial phase, as well as when the semiology evolved during the follow-up.

## Case presentation

A 43 year-old right-handed woman with medical history of episodic depression presented with transient memory and attentional deficits after an episode of a gastric bleeding. She also reported episodes of lacunar amnesia and another kind of episode with sudden loss of consciousness and hypotonia, lasting for 3 or 4 min, which looked like sleep attacks.

Three months after symptoms-onset, one of these transient episodes induced a car accident with a minor head trauma (normal score in the initial Glasgow Coma Scale). The day after the accident, partial visual loss of the left eye, left tinnitus, partial left hearing loss and weakness of the left arm occurred. and two months later, she developed a speech disorder. She was then referred to the Neurology Departement of Besançon.

She worked in the ordering service of an optician. She was divorced 3 years ago and she lived alone with her two children (14 and 18 years-old). She had a twin brother who committed suicide at 22 years old and one healthy older sister. Her grandmother also committed suicide (hung herself). Neurological examination showed visual deficit mimicking cerebral visual impairment (see below neuropsychological testing), slurred-speech, non anatomical sensory loss including the left face and the left lower limb, and a left weakness with positive clinical signs; Hoover sign and drift without pronation [5, 6], as well as in-coordinate limb movements that were both variable and distractible.

Language examination (by a speech therapist) showed a language disorder with anomia, dysprosodia, spasmodic dysphonia, and stuttering. Picture-naming tests were impossible due to visual impairment which was discordant with the fact that reading and writing were not affected. Neuropsychological assessment showed attentional fluctuation (especially in the visual domain), memory impairment and dysexecutive syndrome with working memory impairment, flexibility disorders, and sensitivity to interference. A complex and atypical cerebral visual impairment was observed with a simultanagnosia, a left unilateral spatial neglect and mental imagery deficit. She only processed local information when presented with a visual scene. As a compensatory strategy, she followed the line of Rey’s figure point by point with her finger and hid some parts of the figure to limit visual interferences to perform the copy. Although during the copy of the Rey complex figure she was not able to process the global information, her reproduction did not display any spatial disorganization.

Psychiatric evaluation showed elements of histrionic personality disorder, such as seduction and immature behavior. Emotional numbing and affective discordance was reported, but no personality test was performed. No depression, hallucination or delusion was observed. She described anxiety symptoms in relation to her accident.

Cerebrospinal fluid (including 14-3-3 protein, oligoclonal bands, Beta-amyloid and Tau protein), blood examination, electroencephalogram, electroneuromyogram, CT, MRI and positron emission tomography (PET) of whole body showed no significant abnormalities. Sleep explorations found an obstructive sleep apnea syndrome with an apnea-hypopnea index of 21.6 per hour. Ophthalmologic and ENT showed no abnormalities.

**Fig. 1:**
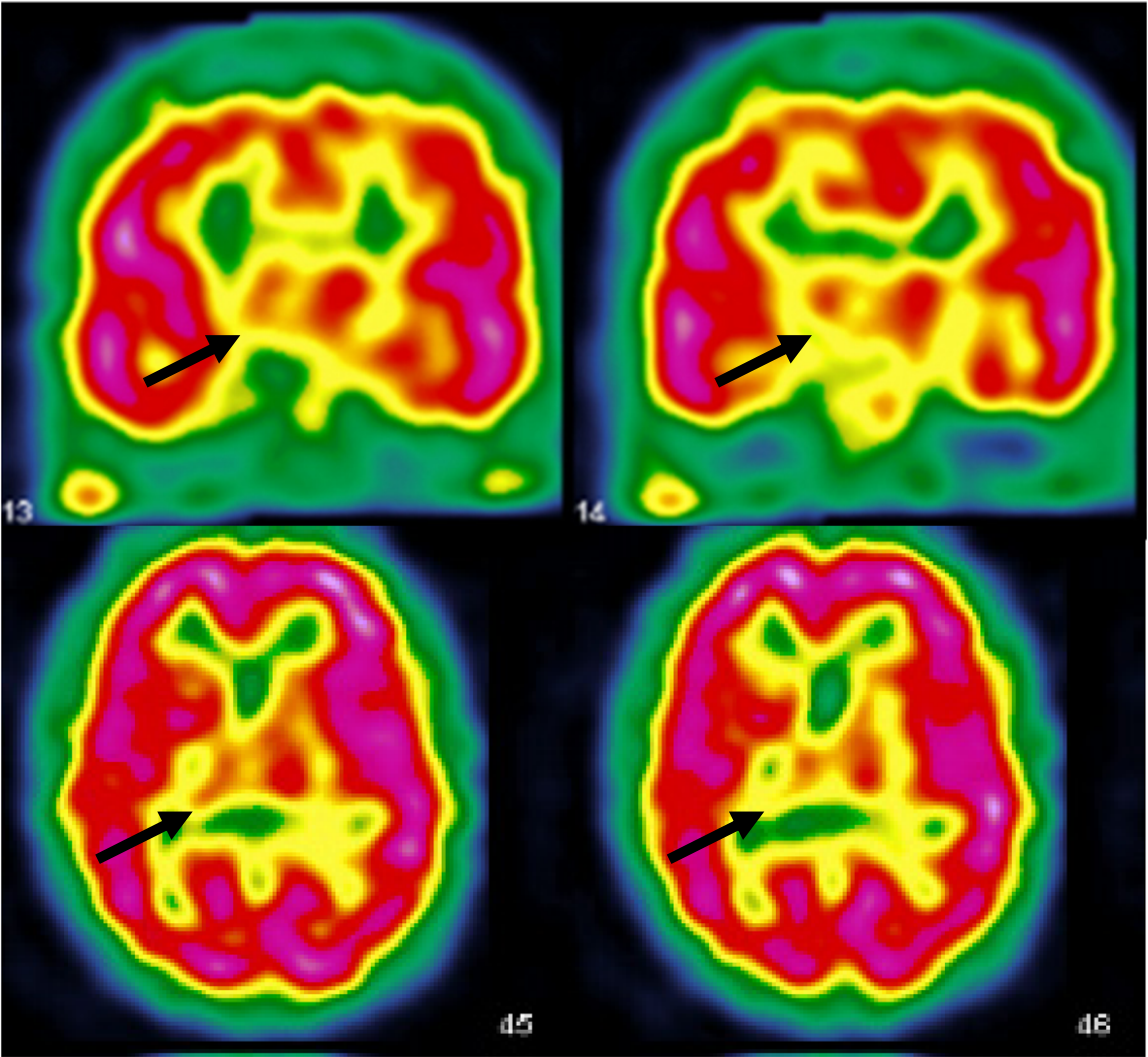
Cerebral SPECT showing a right thalamic hypoperfusion at the initial period of the patient history

Based on the clinical history (abrupt onset after gastric bleeding, waxing and waning illness course with periods of remission) variable neurological symptoms and “positive” clinical signs sush as Hoover, drift without pronation, non-anatomical sensory loss, inconsistence in visual impairment, we made diagnosis of FND according to the DSM-5 criteria. Furthermore, risk factors were present, such as psychological and physical trauma (brother’s suicide and car accident) or elements of personality disorder (immaturity, seduction). The disorder was not attribuable to other rarer entities as an extensive workup was negative, including looking for paraneoplasic and neuroimmunological disorders. A first cerebral single photon emission computed tomography (SPECT, 99mTc-hexamethylpropylene-amineoxime) showed a hypoperfusion of the right thalamus (Fig. 1).

One year after the symptoms onset, the patient presented with atypical ‘blind’ behaviors. She used a cane, but she was able to apply make-up correctly and cleaned her apartment without assistance.

Ophthalmologic assessment with visual acuity, pupil reaction, ocular and fundoscopic examination, optical coherence tomography (OCT) of macula and optic nerve were normal. Static and kinetic visual field perimetric examinations were non interpretable because of the attention deficit. Neurological examination differs from the first one with disappearance of the left sensory loss and the left weakness. A minim subjective left clumsiness persists but very fluctuant. Visuoperceptive impairment was confirmed by speech and neuropsychological assessment. An atypical letter alexia without any deficit for words or sentences reading was observed. To recognize objects, she sometimes needed to manipulate them or she identified them from a detail (identification of the match box when seeing matches). Copying of geometric figure (Rey complex figure test) and tests involving visuospatial and graphomotor skills (crossing of test, Trail Making Test or BEC’s triangle) were not assessable because she reported an unbearable visual discomfort. She avoided seeing some objects, such as a striped shirt, because she reported a too “aggressive” feeling, which is suggestive of a visual intolerance to complex visual patterns and visual fatigability.

**Fig. 2:**
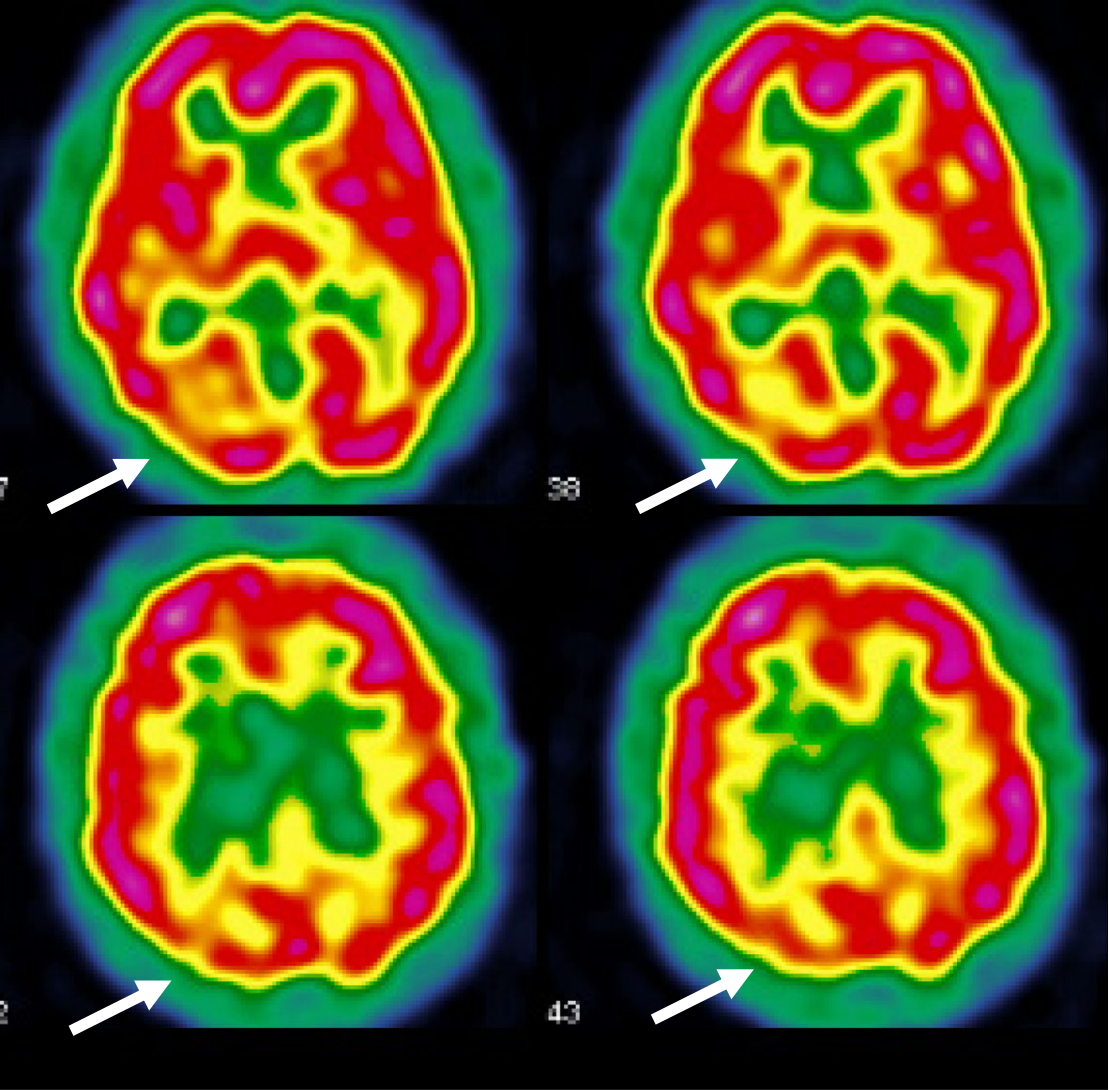
Second cerebral SPECT showing hypoperfusion in the right temporo-occipital junction, right inferior parietal lobe and left superior frontal lobe

A second SPECT 2 years after the symptoms-onset, while she presented with episodes of blindness and atypical cerebral visual impairments, showed hypoperfusion in the right temporo-occipital junction, right inferior parietal lobe, explaining the spatial and attentional deficits, and left superior frontal lobe (Fig. 2).

At our last follow-up (6 years after onset), the visual impairments persisted, as well as a clumsiness of left upper and lower limb. The rest of the examination did not show new clinical signs, which supported our initial hypothesis of FND rather than a progressive neurological disorder.

## Discussion and conclusion

This case illustrates the variable symptomatology encountered in FND with the association of sensorimotor deficits, speech impairment, unilateral spatial neglect, a complex cerebral visual impairment and sleep-like attacks, probably corresponding to PNES. Frequently, FND semiology associates different domains and can vary over time with cognitive, behavioral, affective and even motor events, mainly movement disorders or PNES. This complex semiology, which corresponds to Sydenham’s “chameleon” syndrome, is sometimes difficult to diagnose. Furthermore, coexistence with an other neurological disorder is not uncommon, especially PNES and epilepsy [7]. Recent studies highlighted the importance of “positive” clinical signs, such as Hoover sign, for paresis of lower limb. They are based on internal inconsistency or incompatibility with recognized neurological or medical disease, and show good specificity and reliability [5, 8]. This is in agreement with recent modifications of the DSM-5 regarding the definition of FND, including “positive signs” and the rate of misdiagnosis being very low [9, 10].

The initial semiology of our patient, including sensorimotor deficit, in-coordinate limb movements, slurred speech, sleep-like attacks, visual impairments and unilateral spatial neglect, was suggestive of a right thalamic syndrome [11, 12]. The initial SPECT indeed revealed a right thalamic hypoperfusion, coherent with this initial clinical presentation.

Subsequently, semiology and neuroimaging findings changed. Visual impairments increased, while other symptoms were no longer present, suggesting a posterior cortical syndrome [13]. A second SPECT examination showed a corresponding right occipitoparietal hypoperfusion when the right thalamic abnormalities disappeared.

Usually, paraclinical examinations have to be normal in FND, but functional neuro-imaging can elicit abnormal cerebral pattern activation or metabolism. During the last decades, the number of publications increased; showing a growing interest of the scientific community for this clinical presentation. The first was in the nineties, showing hypoperfusion in the sensorimotor cortex contralateral to a functional hemiparesis [2, 3]. Since then, many functional dysfunctions have been reported in FND in various cortical and subcortical structures, including basal ganglia [14]. The authors reported simultaneous anterior cingulate and medial prefrontal cortex hyperperfusions, suggesting an increased inhibition of the motor cortex, probably under influence of the limbic system, including motivational and emotional processes. Basal ganglia seem to also play a key role in FND. The physiological role of basal ganglia in the elaboration of the movement was found to be modified during FND, with an exaggerated monitoring of the limbic system interfering with motor pattern [15, 16]. Numerous hypotheses emerged from neuroimaging studies, such as emotional dysregulation, memory suppression, and abnormal self-monitoring or sense of agency [17–22]. Mechanism of dissociation have also been suggested to play role in FND [1, 23, 24]. Dissociation is a process in which specific functions (such as memory or sensation) are disrupted and no longer integrated into conscious unified processes. Neuroimaging research in the field of dissociation also highlighted the role of similar brain structures implicated in emotion regulation such as prefrontal region [25, 26].

However, to date no definite universal abnormal neuroimaging pattern has been identified in FND. This is probably explained by the complexity of this disorder and the polymorphic clinical presentation. These findings support Jean-Martin Charcot’s point of view (1825–1893). He developed the concept of a “psychodynamic” lesion which is invisible in a macroscopic way and which can evolve, resulting in a varied symptomatology. A previous study showed modification of cerebral perfusion with an hypoperfusion in the thalamus and basal ganglia contralateral to functional sensorimotor deficit which disappeared after clinical recovery [16]. Our case similarly illustrates an evolving and dynamic lesion with a variable neuroimaging pattern. First we had a right thalamic hypoperfusion concordant with right thalamic syndrome and then, later, an evolving semiology with predominant neurovisual disorders with a concordant hypoperfusion in the occipito-parietal cortex.

Another originality of our report is the unusual complex central neurovisual disorders, suggesting posterior cortical dysfunction, which occurred during the follow-up [13]. To our knowledge, this is the first report of a higher visual dysfunction of dorsal and ventral pathways in functional visual disorders (FVD), instead of the classical loss of acuity or a vision field loss. FVD represents 1–5% of patients in ophthalmology clinics with loss of acuity, visual field abnormalities, diplopia, oscillopsia, ptosis and blepharospasm [27]. The most frequent complaints are monocular vision loss, concentric loss of peripheral vision and spasm of near reflex in motor ocular disorder. They share the same demographic characteristics with other FNDs.

One study compares cerebral pattern activation of FVD versus healthy subjects and showed a reduced activation in the primary visual cortex, with increased activation in the posterior cingular cortex, basal ganglia, insula, and prefrontal cortex [4]. This pattern differs from patients with neuro-ophthalmologic lesion [28]. Another study do not find FVD patterns in neuroimaging, but rather a neurophysiological modification of N1 event-related potential response during blindness that disappeared after recovery [29].. N1 response comes from the intraparietal sulcus and plays a role in spatial sustained attention to locate stimuli in the visual peripheral field. Misused dysfunction of these mechanisms may be involved in FVD. In our case, the fatigability and discomfort in front of complex visual stimuli, such as visual patterns, may correspond to visual attention disorders inducing visual detection deficit. Abnormal top-down control for spatial attention could contribute to the deficit, which is supported by hypoperfusion in occipito-parietal cortex seen on SPECT in our case.

Our patient presented with simultanagnosia and abnormalities seen in the right parietal lobe, which is concordant with the “dorsal stream” of the visual processing network [30, 31]. It is involved in the treatment of spatial data and movement. Its dysfunction leads to Balint syndrome including ataxia, ocular motor apraxia and simultanagnosia [32].

Our patient presented with visual associative agnosia, with preserved forms and color descriptions, but difficulties in the identification of visual objects requiring the use of another sensorial modality (tactile modality) to recognize items [33]. Furthermore, she also presented with an atypical alexia without agraphia, which is frequently associated with visual agnosia. Alexia was considered as atypical because the deficit was on preponderant letter recognition and less impaired for words, which is usually the opposite (words more impaired than letters) or both items are impaired. Alexia without agraphia involved the left occipitotemporal pathway, including the visual word form area in the left fusiform gyrus specialized in word form recognition. The occipito-temporal hypoperfusion observed in our case was quite concordant with this agnosic and alexic semiology with a quite preserved left occipitotemporal pathway, which may explain the preserved word recognition. All these abnormalities lead to a dysfunction in the “ventral stream” of the visual processing, which is involved in the identification of objects [30, 31].

Interestingly, our patient worked in an ordering service of an optician and developed neurovisual symptoms. According to Jean Martin Charcot and Pierre Janet, the notion of suggestibility is closely related to FND history. For a long time, social and cultural process was considered as a huge influencing factor in the ethiopathogenesis of FND and high degree of suggestibility seemed to be a characteristic. Other studies showed the opposite and the distinction between feigners and FND is not easy [34–36].

Our case report has several limitations: obviously a discussion based on a single subject study should be handle with caution. The SPECT could be over-readed considering non-specific hypoperfusions but anatomoclinical concordance in term of laterality and cortical and subcortical structures support our findings. Secondly, our neuropsychological evaluation did not include formal symptom validity test (SVT) and our interpretation that it corresponded to a functional disorder was only based on our clinical experience [37]. Third, an effect of medication on our neuroimaging findings is possible, even if localized changing hypoperfusion are not typical for medication-induced neuroimaging changes.

In conclusion, our report highlights 1/ the evolving neuroimaging patterns concordant with the variable semiology of FND, suggesting the charcot’s “psychodynamic lesion”, 2/ the possibility of complex central neurovisual disorders suggesting posterior cortical dysfunction of dorsal and ventral pathways instead of more primary visual disorders (loss of acuity or visual field defect) in FVD and 3/ the potential role of functional neuroimaging as biomarkers in FND.

## Data Availability

All data generated or analysed during this study are included in this published article.

## Abbreviations

CT: Cerebral tomodensitometry
DSM: Diagnostic and statistical manual of mental disorders
FND: Functional neurological disorder
FVD: Functional visual disorder
ICD: International classification of diseases
MRI: Magnetic resonance imaging
PET: Positron emission tomography
PNES: Psychogenic non-epileptic seizure
SPECT: Single photon emission computed tomography
SVT: Symptom validity test
